# Narrative Review on the Management of Neck of Femur Fractures in People Living with HIV: Challenges, Complications, and Long-Term Outcomes

**DOI:** 10.3390/microorganisms13071530

**Published:** 2025-06-30

**Authors:** Yashar Mashayekhi, Chibuchi Amadi-Livingstone, Abdulmalik Timamy, Mohammed Eish, Ahmed Attia, Maria Panourgia, Dushyant Mital, Oliver Pearce, Mohamed H. Ahmed

**Affiliations:** 1 Department of Orthopaedic, Leicester University Hospital, Leicester LE1 5WW, UK; yashar.mashayekhi1@nhs.net; 2 Department of Emergency Medicine, York and Scarborough NHS Foundation Trust, York YO31 8HE, UK; chibuchi.amadi-livingstone@nhs.net; 3 Department of Trauma and Orthopaedic, Milton Keynes University Hospital NHS Foundation Trust, Eaglestone, Milton Keynes MK6 5LD, UK; abdulmalik.timamy@mkuh.nhs.uk (A.T.); mohammed.eish@mkuh.nhs.uk (M.E.); ahmed.attia@mkuh.nhs.uk (A.A.); oliver.pearce@mkuh.nhs.uk (O.P.); 4 Department of Geriatric Medicine, Milton Keynes University Hospital NHS Foundation Trust, Eaglestone, Milton Keynes MK6 5LD, UK; maria.panourgia@mkuh.nhs.uk; 5 Faculty of Medicine and Health Sciences, University of Buckingham, Buckingham MK18 1EG, UK; 6 Department of Blood Borne Virus and HIV, Milton Keynes University Hospital NHS Foundation Trust, Eaglestone, Milton Keynes MK6 5LD, UK; dushyant.mital@mkuh.nhs.uk; 7 Department of Medicine and HIV Metabolic Clinic, Milton Keynes University Hospital NHS Foundation Trust, Eaglestone, Milton Keynes MK6 5LD, UK

**Keywords:** HIV, femur neck fractures, bone mineral density, antiretroviral therapy, systematic review

## Abstract

Neck of femur (NOF) fractures are a critical orthopaedic emergency with a high morbidity and mortality prevalence, particularly in people living with Human Immunodeficiency Virus (PLWHIV). A combination of HIV infection, combined antiretroviral therapy (cART), and compromised bone health further increases the risk of fragility fractures. Additionally, HIV-related immune dysfunction, cART-induced osteoporosis, and perioperative infection risks further pose challenges in ongoing surgical management. Despite the rising global prevalence of PLWHIV, no specific guidelines exist for the perioperative and post-operative care of PLWHIV undergoing NOF fracture surgery. This narrative review synthesises the current literature on the surgical management of NOF fractures in PLWHIV, focusing on pre-operative considerations, intraoperative strategies, post-operative complications, and long-term outcomes. It also explores infection control, fracture healing dynamics, and ART’s impact on surgical outcomes while identifying key research gaps. A systematic database search (PubMed, Embase, Cochrane Library) identified relevant studies published up to February 2025. Inclusion criteria encompassed studies on incidence, risk factors, ART impact, and NOF fracture outcomes in PLWHIV. Data were analysed to summarise findings and highlight knowledge gaps. Pre-operative care: Optimisation involves assessing immune status (namely, CD4 counts and HIV-1 viral loads), bone health, and cART to minimise surgical risk. Immunodeficiency increases surgical site and periprosthetic infection risks, necessitating potential enhanced antibiotic prophylaxis and close monitoring of potential start/switch/stopping of such therapies. Surgical management of neck of femur (NOF) fractures in PLWHIV should be individualised based on fracture type (intracapsular or extracapsular), age, immune status, bone quality, and functional status. Extracapsular fractures are generally managed with internal fixation using dynamic hip screws or intramedullary nails. For intracapsular fractures, internal fixation may be appropriate for younger patients with good bone quality, though there is an increased risk of non-union in this group. Hemiarthroplasty is typically favoured in older or frailer individuals, offering reduced surgical stress and lower operative time. Total hip arthroplasty (THA) is considered for active patients or those with pre-existing hip joint disease but carries a higher infection risk in immunocompromised individuals. Multidisciplinary evaluation is critical in guiding the most suitable surgical approach for PLWHIV. Importantly, post-operative care carries the risk of higher infection rates, requiring prolonged antibiotic use and wound surveillance. Antiretroviral therapy (ART) contributes to bone demineralisation and chronic inflammation, increasing delayed union healing and non-union risk. HIV-related frailty, neurocognitive impairment, and socioeconomic barriers hinder rehabilitation, affecting recovery. The management of NOF fractures in PLWHIV requires a multidisciplinary, patient-centred approach ideally comprising a team of Orthopaedic surgeon, HIV Physician, Orthogeriatric care, Physiotherapy, Occupational Health, Dietitian, Pharmacist, Psychologist, and related Social Care. Optimising cART, tailoring surgical strategies, and enforcing strict infection control can improve outcomes. Further high-quality studies and randomised controlled trials (RCTs) are essential to develop evidence-based guidelines.

## 1. Introduction

The prognosis for individuals with HIV has greatly improved in recent decades as a result of advancements in combined antiretroviral therapy (cART). The implementation of highly active antiretroviral therapy (HAART) in the mid-1990s was a pivotal moment, significantly decreasing the rates of illness and death primarily linked to complications of HIV infection [1]. Consequently, people living with Human Immunodeficiency Virus (PLWHIV) now have a life expectancy that is similar to that of the general population. This has led to a change in therapeutic focus towards the management of long-term non-communicable health conditions that are frequently associated with ageing [2]. Among all these concerns, one that stands out is bone health, specifically the risk of fractures of the neck of the femur (NOF). These fractures are particularly concerning since they are associated with high levels of morbidity and mortality [3].

NOF fractures are especially important in PLWHIV because they are influenced by the intricate relationship between bone mineral density (BMD) loss associated with HIV and the potential problems caused by cART. Chronic HIV infection is associated with persistent systemic inflammation and immunological activation, both contributing to the acceleration of bone loss and reduction in BMD [4]. Furthermore, certain cART regimens, particularly those that include Tenofovir Disoproxil Fumarate (TDF), have been strongly linked to additional decreases in BMD and an elevated likelihood of developing osteoporosis and experiencing further widespread fractures [5]. As a result, PLWHIV are more likely to be afflicted by non-osteoporotic NOF fractures compared to the general population. These fractures have a substantial impact on both the clinical and economic aspects of their lives.

The surgical treatment of NOF fractures in PLWHIV poses distinct but potentially manageable difficulties. As HIV per se and cART-related variables frequently experience decreased bone quality, which can affect the process of surgical repair and healing [6]. Moreover, the heightened likelihood of post-operative problems, such as wound-related infections and delayed healing, requires a tailored strategy for both the surgical procedures involved and the subsequent treatment and management post-operatively [7]. Recent research indicates that although traditional surgical procedures like hip arthroplasty or internal fixation are still often used, there is an increasing demand for adjustments in surgical planning and post-operative care to address the unique risks faced by PLWHIV [8]. Considering the growing prevalence of NOF fractures in ageing individuals living with HIV, it is crucial to thoroughly analyse the most recent evidence-based studies regarding the factors that contribute to the risk of these fractures, the surgical methods used to treat them, and the care provided after the surgery. This study review intends to analyse existing evidence in order to identify PLW HIV who are at the highest risk for the fracture NOF. Additionally, it attempts to suggest specific surgical and non-surgical therapies that can reduce this risk and enhance patient outcomes.

## 2. Methodology

This study is a narrative review that collects and presents current research on the surgical treatment of NOF fractures in PLWHIV infection. A structured literature search was conducted using electronic databases, including PubMed (MEDLINE), Embase, Cochrane Library, Google Scholar, and Web of Science. The search strategy included a combination of Medical Subject Headings (MeSH) terms and free-text terms to capture all possible relevant studies.

The primary search terms included “neck of femur fracture”, “hip fracture” “HIV”, “people living with HIV”, “HIV positive” “Surgical management”, “operative treatment”, “hip arthroplasty”, “osteoporosis”, “bone health”, “fragility fractures”, “post-operative complications”, “infection risk”, and “perioperative care” in an attempt to obtain the desired results in a wide range of scenarios.

The final search was limited to English language publications only. Using the same eligibility criteria, in this review, studies were identified based on the following inclusion and exclusion criteria: Inclusion Criteria:

Peer-reviewed studies published in English; research conducted on NOF fractures in PLWHIV articles that describe surgical procedures; the management of the patient during the operative period and the results after surgery; research on osteoporosis, fragility fractures, and the effects of cART on bone health. Studies were restricted to those published between 2000 and 2025.

Exclusion Criteria

**Figure 1:**
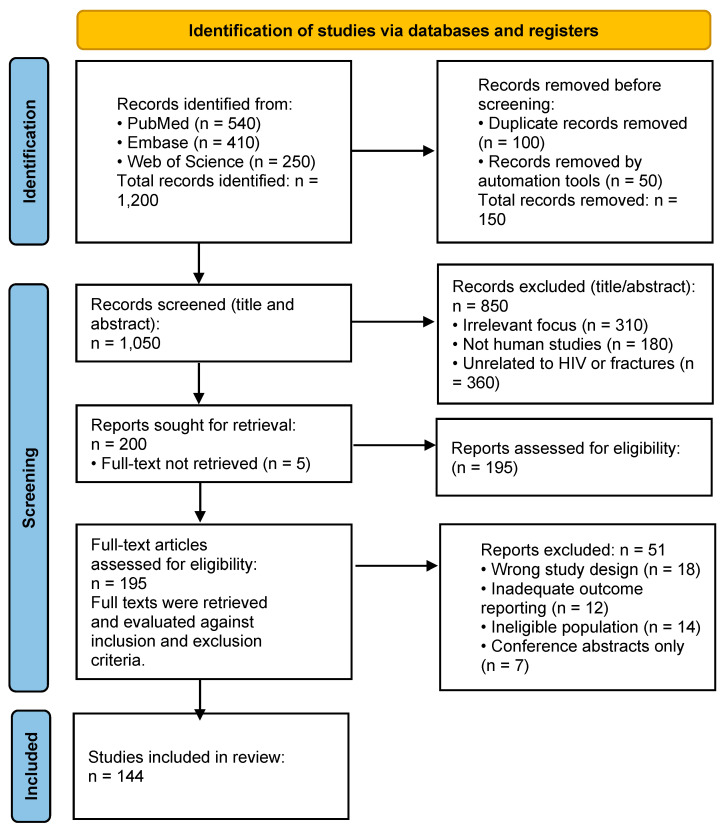
PRISMA diagram showing steps taken to conduct this literature review.

Research that was conducted using participants who did not have HIV; studies that focused on the management of NOF fractures without surgery or where surgery was used as a part of conservative management; low evidence case reports; publications in languages other than English (Figure 1).

## 3. Epidemiology

There is growing evidence that people living with HIV (PLWHIV) are at an increased risk of bone fractures. For example, PLWHIV are twice as likely as HIV-negative people to sustain fractures, including those in the NOF [9]. Furthermore, the HIV Outpatient Study (HOPS) data from the United States revealed that PLWHIV had a higher frequency of fractures than the general population [10]. Similarly, a separate population-based cohort study found a higher incidence of NOF fractures in PLWHIV [11]. These conclusions highlight the value of targeted preventative efforts in this particular demographic. Additionally, numerous regional studies have also reported variations in fracture incidence among PLWHIV. For example, a South African cohort study showed a much higher prevalence of NOF fractures in PLWHIV than in the general population. This incidence was connected to the high HIV prevalence and widespread use of TDF-based regimens, which have been demonstrated to have an impact on bone health [12]. Moreover, research in Asia shows that the prevalence of NOF fractures among PLWHIV is increasing, particularly in countries with ageing HIV-infected populations. Shao et al. showed in a study conducted in China a higher prevalence of low BMD in PLWHIV, especially those above the age of 50 years and on treatment with lopinavir/ritonavir [13]. These figures highlight the crucial need for a better understanding of bone health and management in the PLWHIV [14,15,16,17]. Preventive measures, such as lifestyle changes, dietary supplementation, and proper cART regimen selection, are critical for lowering fracture risk in this vulnerable population [18,19,20,21]. Multiple studies have demonstrated that people living with HIV (PLWHIV) are at significantly higher risk of fragility fractures, including neck of femur (NOF) fractures, compared to HIV-negative individuals of similar age [22,23,24,25,26,27]. A nationwide Danish case-control study found that HIV infection was associated with a threefold increased risk of all clinical fractures and an odds ratio of 8.99 for hip fractures, independent of age and sex [28]. Similarly, the Veterans Ageing Cohort Study reported an incidence rate of 2.6 per 1000 person-years for fragility fractures among HIV-positive men, which was higher than in the general population, highlighting the elevated burden of fractures in this group [29]. A subsequent study from the same cohort also identified frailty, polypharmacy, and prior falls as significant fracture predictors, even after adjusting for HIV status [30]. Furthermore, a large systematic review and meta-regression, including over 32,000 PLWHIV, found that the overall risk of fracture in HIV-positive individuals was approximately twice as high as in matched HIV-negative controls [22]. These findings support the conclusion that age-matched comparison alone underestimates the fracture burden in PLWHIV, with additional contributions from chronic inflammation, cART exposure, and HIV-related comorbidities (Table 1).

This greater risk of fracture occurs with the age-related changes in HIV populations and is observed approximately 10 years earlier than in the general population [18,19]. As a result of the ageing process, the bone deteriorates in composition, structure, and function, which predisposes individuals to osteoporosis. A combination of genetic, hormonal, biochemical, and environmental factors underlies the pathophysiology, resulting in marked bone loss and, therefore, increased fracture risk [20] (Table 2). In people living with HIV, this risk is further exacerbated due to the virus itself and the long-term use of an ART, which can lead to bone demineralisation, thereby causing osteoporosis and increasing susceptibility to femoral neck fractures [21]. A meta-analysis that assessed over 32,000 patients found older age (>60) to be a statistically significant risk factor for hip fracture among people living with HIV [22].

Women are more susceptible to osteoporosis and are, therefore, at an increased risk of fracture compared to their male counterparts, with one study finding that the prevalence of femoral neck fractures is three times higher in women [23]. This increased risk in women can be attributed to several factors, particularly hormonal changes that are prevalent in postmenopausal women. A systematic review found that menopause is an important predictor of bone loss and low bone mineral density in HIV-infected postmenopausal women; however, it states that there is still inadequate evidence to suggest a role in fracture rates at this time [24]. A multicentre prospective cohort study found that in premenopausal women, fracture rates were not significantly greater than in HIV-uninfected women [25]. Similarly, a study in 2013 stated that fracture rates increased to a greater extent in women than in men, and this increase is shown to occur as anticipated at the time of the perimenopausal transition [23]. Some other factors, such as white race and alcohol abuse, have been identified as increasing fracture risk in HIV-infected individuals [26,27] (Table 2). Importantly, people living with HIV (PLWHIV) have a higher incidence of neck of femur (NOF) fractures compared to the general population, and this risk increases with the duration of HIV infection. Studies suggest that the risk of fractures rises significantly in PLWHIV over the age of 50. In Table 2, we have also explained other factors associated with fracture NOF. The relationship between cART and fracture risk in PLWHIV is complex. While cART improves overall survival and reduces HIV-related complications, it may also contribute to bone mineral density (BMD) loss, particularly in the early phases of treatment. Several longitudinal studies and meta-analyses have shown that individuals initiating cART experience rapid declines in BMD within the first 6–12 months, with tenofovir disoproxil fumarate (TDF) being most strongly implicated in this bone loss [5,31,32,33]. One large prospective study reported that fracture rates increased after starting cART and were most pronounced in those receiving TDF or protease inhibitors [34]. Conversely, individuals who are cART-naïve or poorly adherent may also have increased fracture risk due to uncontrolled viraemia, chronic inflammation, vitamin D deficiency, and secondary effects of immunosuppression, such as frailty or opportunistic infections. The START Bone Mineral Density substudy found that ART-naïve individuals had higher baseline BMD, but BMD declined rapidly following ART initiation, although it then plateaued with ongoing therapy [35]. Thus, both untreated HIV and ART exposure can independently contribute to reduced bone health. These findings highlight the need for careful ART regimen selection, early screening for osteoporosis, and proactive bone health management—particularly in older patients or those with multiple risk factors. Epidemiological studies indicate that people living with HIV (PLWH) have a higher risk of fractures, including neck of femur (NOF) fractures, compared to HIV-negative individuals. However, the specific role of high HIV viral load in increasing fracture risk is less clearly defined. A nationwide case-control study in Denmark found that HIV infection was associated with a threefold increased risk of fractures, independent of age and sex. Notably, the odds ratio for hip fractures was 8.99, highlighting a significant association between HIV and NOF fractures [28]. Similarly, a study from the Veterans Ageing Cohort Study (VACS) reported an incidence rate of fragility fractures, including hip fractures, of 2.6 per 1000 person-years among HIV-positive men. This rate was higher than that observed in the general population, suggesting an elevated fracture risk in PLWHIV. While the overall association between HIV and increased fracture risk is well-documented, the direct impact of high viral load on fracture risk is less clear [29,36]. Some studies have not found a significant association between viral load and bone mineral density (BMD) or fracture risk. For instance, the START Bone Mineral Density Sub study did not observe a significant relationship between HIV viral load and BMD in ART-naïve individuals [35]. In conclusion, while high HIV viral load may contribute to mechanisms that increase fracture risk, more research is needed to establish definitive evidence. Nonetheless, the overall increased risk of fractures in PLWHIV underscores the importance of proactive bone health management in this population. Besides viral load, other factors are also important to consider. For example, a study analysing 21,041 older persons living with HIV (PWH) on ART from the Veterans Ageing Cohort Study (2010–2015) found that physiologic frailty, measured by the VACS Index 2.0, was a significant risk factor for both serious falls (15%) and fragility fractures (13%). Other modifiable risk factors included a serious fall in the past year, polypharmacy, recent opioid prescriptions, and alcohol use disorder. These findings highlight the importance of targeting these factors in prevention programs to reduce the incidence of serious falls and fragility fractures among older PLWHIV [30].

**Table 2:** Provides summary of different factors that contribute to the pathogenesis leading to NOF in PWLHIV. Different factors can contribute to the complex process of osteoporosis.

Mechanism	Details	Impact on Bone Health	References
Chronic Inflammation and Immune Activation	Triggers TNF-α and IL-6 release.	Increases osteoclast activity and bone loss.	[37]
Decreased Osteoblast Function	Reduces osteoblast activity and apoptosis	Impaired bone formation, increasing fracture risk.	[38,39]
Chronic Immune Activation Despite cART	Ongoing inflammation persists despite cART.	Decreases bone turnover, increasing fracture risk.	[4,40]
Tenofovir Disoproxil Fumarate (TDF)	Use in treatment of HIV	TDF associated with decreased bone mineral density (BMD) and increased osteoporosis risk due to phosphate deficiency (due to renal loss) and altered vitamin D metabolism	[31,32,33]
Protease Inhibitors (PIs)	Use in treatment of HIV	PIs impair osteoblast and osteoclast function, interfering with normal bone remodelling processes	[34]
Mitochondrial Toxicity from cART	Mitochondrial Toxicity may contribute to process of osteoporosis	cART, particularly Protease Inhibitors, can cause mitochondrial toxicity and osteoblast death that further reduces bone synthesis, thereby impairing bone health.	[41]
Hepatitis C Co-Infection	HCV in HIV patients affects liver function.	Low vitamin D and calcium absorption, increasing osteoporosis risk.	[42,43]
Other Medical Conditions (CKD, Diabetes, Hypogonadism)	HIV patients often have CKD, diabetes, or hormonal imbalances due to cART.	CKD: Alters calcium/phosphate balance, worsening bone loss. Diabetes: Reduces bone quality, increasing fracture risk. Hypogonadism: Low testosterone/oestrogen worsens osteoporosis.	[41,42,43,44,45]
Gender-Specific Risks	Premature ovarian failure may increase risk of osteoporosis	Postmenopausal women are particularly vulnerable to fractures. Gender-specific interventions are necessary.	[46,47,48]
Smoking and alcohol	High prevalence of smoking	Smoking, alcohol use, and low body weight exacerbate fracture risk.	[49,50]
Economic, social, and dietary factors	NOF fractures are exacerbated by financial strain, social barriers, and nutrient deficiencies linked to cART and malabsorption.	NOF fractures lead to increased medical costs, longer hospital stays, and reduced quality of life. Long-term indirect costs are often underrepresented in economic evaluations.	[51]

### 3.1. Social Factors Associated with HIV and Their Detrimental Impact on NOF

Social factors, including isolation, depression, living alone, and a lack of support, also affect the management of bone health in PLWHIV, both in the general population and in the post-operative period. Social isolation and depression may lead to non-adherence to all treatment protocols and delayed health care seeking, with consequent increase in the risk of fractures and slow healing after surgery. These psychosocial problems are of particular importance for PLWHIV since they can result in the inadequate management of both HIV and other comorbidities, including bone diseases [52,53]. Patients’ post-operative care is fraught with potential difficulties in terms of management if they live alone and have a lack of carers. This includes non-compliance with medication, inability to perform rehabilitation exercises and non-attendance for scheduled follow-up appointments. Such steps are important for the healing process after femur neck fractures, and compliance has good outcomes [54,55]. Studies have established that social support is a good indicator of health outcomes. Low social support reduces the probability of positive outcomes and increases the chances of negative results following surgery [56]. These psychosocial factors are, therefore, important to consider in order to provide total care to enhance the positive results and outcomes for patients with NOF fractures. These considerations should be incorporated into the routine clinical practice with the relevant stakeholders, which would further enable a more comprehensive management of bone health in this at-risk population. Multidisciplinary interventions may involve pharmacological and lifestyle changes, as well as psychological and social interventions, such as counselling, community support, and rehabilitation services [57,58]. There is a high likelihood that patients will revert to the same environment and the same challenges post-operatively if they are discharged back home without any assistance. Thus, it is important to offer patients continuous support in order to discontinue the cycle of poor outcomes and potentially enhance long-term recovery rates. It is necessary to develop a comprehensive plan for the prevention and management of NOF fractures in PLWHIV. This strategy should include BMD scanning, potentially recommending lifestyle changes, optimising cART regimens, and potentially initiating new treatment strategies. Through this work, healthcare providers can improve the health and quality of life for PLWHIV.

### 3.2. NOF Fractures in PLWHIV: Addressing the Challenge in Developing Countries

**Figure 2:**
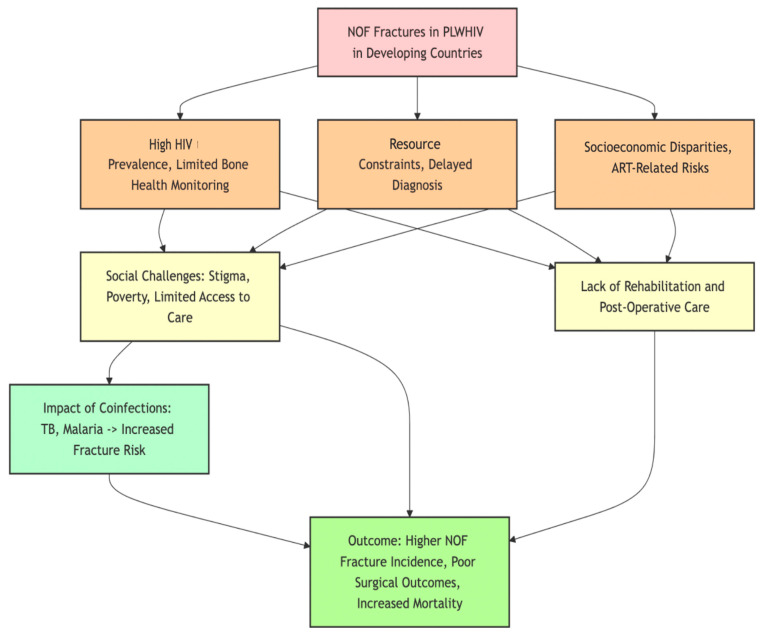
Demonstrating factors affecting treatment of NOF fractures in PLWHIV in developing countries.

The incidence of NOF fractures in PLWHIV is not constant and varies across the world, with resource-limited countries in Africa, South Asia, and South America having certain healthcare-related challenges. The affected regions are characterised by high HIV prevalence and limited health facilities. This is a different epidemiological situation compared to high-income countries, and the consequences of the management of NOF fractures in these places are often varied. In the sub-Saharan regions of Africa, there has been a steady rise in the incidence of fractures among PLWHIV. This can be attributed to the fact that HIV is a prevalent disease in the region, and there is also an increasing incidence of osteoporosis as a result of the chronic HIV infection. Evidence suggests that in South Africa, a country with approximately 7.5 million people living with HIV, there has been a rising trend of osteoporotic fractures, particularly NOF fractures, among the elderly HIV-positive individuals [19,59,60]. This trend is further exaggerated by the fact that many people are on TDF as part of cART, which is a typical HIV drug that has been associated with lower BMD [61]. India, which has the largest HIV epidemic and a growing elderly population, is also seeing rising NOF fracture numbers in PLWHIV because of the lack of access to Dual-energy X-Ray Absorptiometry (DEXA) scans, which are critical in assessing BMD. Any delay in this diagnosis and further management of the condition can potentially further increase the risk of fractures among this particular cohort [62]. Another critical issue is that bone health check-ups are not performed routinely in HIV clinics; thus, PLWHIV may go undiagnosed and untreated for osteoporosis. Brazil is noted for having high HIV prevalence and an increasing incidence of NOF fractures. Research shows that PLWHIV in Brazil are at increased risk of bone fractures. This increased risk is due to both the decrease in BMD that is seen with the use of cART and other lifestyle factors such as smoking and low levels of physical activity [63]. The situation is further complicated by socioeconomic inequalities that limit access to quality HIV and osteoporosis care. Social challenges in relation to NOF fractures in PLWHIV in resource-limited countries are extensive. In many of the African and South Asian societies, HIV-related stigma is still high, and people tend to delay seeking specialised HIV-related health care and not adhere to the medications prescribed to them [64]. In addition, the constraints in these areas mean that PLWHIV have to focus on their basic needs rather than on the management of their health and preventing or managing osteoporosis. It is established that some rural areas of Africa and South Asia lack the facilities that can manage complex fractures like NOF fractures, resulting in many patients failing to have the surgery they require. This has the consequence of extended disability and higher rates of mortality [65,66]. These consequences are worsened by the lack of post-operative care and rehabilitation services, as many patients do not have the chance to have the follow-up care and physical therapy that is crucial for the healing of NOF fractures (Figure 2). Research has also lately established that in some tropical areas, HIV-related NOF fractures may be worsened by co-infections with endemic diseases like Tuberculosis (TB) and Malaria, which is not uncommon in resource-limited countries. For instance, TB is associated with direct bone infection (osteomyelitis) and secondary bone loss, resulting from prolonged inflammation and use of corticosteroids. This is because TB affects bone health and increases the fracture risk among PLWHIV [67]. The tropical diseases and their effect on bone health in PLWHIV is an interesting and growing area of research. Moreover, malnutrition, which is a major problem in many developing nations, worsens bone health and increases the risk of NOF fractures in PLWHIV [15]. The combination of malnutrition, inflammation, and low BMD due to cART treatment creates an unfavourable environment for fractures in these particular populations. In addition, there is a high prevalence of diabetes, dyslipidaemia, metabolic syndrome, and CKD that will not receive early clinical attention [68,69].

Hence, it is crucial to offer specific treatments for these patients that aim at improving their nutritional status and bone density. Therefore, the management of NOF fractures in PLWHIV in developing nations requires a holistic strategy considering the specific socio-economic, cultural, and health system challenges in these contexts. To address these fractures effectively, it is important not only to improve the accessibility of surgical care and post-operative rehabilitation but also to integrate bone health screening into routine HIV care. Furthermore, continued research is needed to determine the effect of other factors, such as tropical diseases, on the incidence of NOF fractures in these populations (Figure 2).

### 3.3. Surgical Techniques for Treating Fractures of the Neck of the Femur (NOF) in Patients with HIV

The summary of surgical techniques used in the management of NOF in PLWIHIV is listed in Table 3, with a detailed explanation of the advantages and disadvantages. There are three main surgical treatments available for treating neck of femur fractures in HIV patients: Internal fixation (dynamic hip screw/cannulated screws), hemiarthroplasty, and total hip arthroplasty (THA). Each of these techniques has its own unique challenges and advantages.

Despite the fact that Cannulated screws are a common internal fixation method and can be used in PLWHIV, some studies suggest a potential for delayed fracture healing and non-union. It can be a suitable choice for younger patients with good bone density [70,71]. Nevertheless, the weakened bone integrity linked to prolonged cART, specifically tenofovir disoproxil fumarate (TDF), presents a significant obstacle (Table 3).

Hemiarthroplasty provides a well-balanced solution for patients who are HIV-positive by minimising the duration and impact of surgery, which is especially advantageous for individuals with weakened immune systems [72]. Nevertheless, HIV-positive patients have a higher likelihood of developing periprosthetic joint infection (PJI), which requires the application of antibiotic-impregnated cement and a period of antibiotic prophylaxis. There is limited direct evidence specifically addressing prophylactic antibiotic use for NOF fractures in people living with HIV. However, general surgical guidelines recommend administering prophylactic antibiotics to reduce the risk of surgical site infections in all patients undergoing orthopaedic procedures, including those with HIV. For individuals with HIV, additional considerations include their immune status, CD4 count, viral load, and any comorbid conditions. In the absence of specific guidelines for HIV-positive patients with NOF, clinicians typically follow standard prophylactic protocols, adjusting as necessary, based on the patient’s overall health and immune function.

For the most current and detailed recommendations, consulting the latest guidelines from orthopaedic and infectious disease societies, as well as recent peer-reviewed studies, is advisable.

Furthermore, the extended longevity of the prosthesis is a matter of worry, particularly in younger individuals who are HIV-positive and may surpass the anticipated lifespan of the implant [73]. Post-operative care should involve diligent surveillance for indications of infection and possible loosening of the implant and acetabular bone erosion. THA is a more comprehensive surgical procedure that entails the replacement of both the femoral head and the acetabulum [74].

It is recommended for those with severe osteoarthritis or displaced femoral neck fractures, especially those who are physically active and have a longer projected lifespan. THA offers superior functional outcomes and pain relief compared to other options [75]. However, it is the most invasive choice, involving a longer surgical intervention and a lengthier recovery period. HIV-positive patients who are undergoing THA have a higher likelihood of experiencing post-operative problems, such as deep infections and fractures around the artificial joint [76]. This risk is particularly elevated in individuals with low CD4 counts or those who do not follow cART well [77]. Importantly, in PLWHIV, fracture-related infections (FRIs) following neck of femur fractures are associated with a distinct microbiological profile. Staphylococcus aureus, including both methicillin-sensitive (MSSA) and methicillin-resistant (MRSA) strains, is a common isolated pathogen, with MRSA occurring 6–18 times more frequently in HIV-positive patients. Coagulase-negative staphylococci, such as Staphylococcus epidermidis, are prevalent, particularly in implant-associated infections due to their capacity for biofilm formation. Gram-negative organisms, including *Escherichia coli*, *Klebsiella* spp., and *Enterobacter* spp., are frequently implicated and often necessitate broad-spectrum antimicrobial therapy. Opportunistic pathogens, such as Pseudomonas aeruginosa and enterococci (*E. faecalis*, *E. faecium*), also contribute, particularly to polymicrobial infections. The increased susceptibility to FRIs in this population is primarily due to immunosuppression, especially in the context of low CD4 counts or inadequate antiretroviral therapy. Empirical antibiotic regimens should be informed by the higher prevalence of MRSA and resistant Gram-negative bacteria to optimise clinical outcomes [78]. When deciding between cemented and uncemented prostheses, it is important to consider the patient’s bone quality. Research indicates that cemented prostheses may provide better initial stability in osteoporotic bone, but they may be problematic if there is significant bone loss at the implant–bone interface [79].

**Table 3:** Demonstrating the surgical options for NOF fractures and their advantages/disadvantages in consideration of PLWHIV.

Surgical Option	Advantages	Challenges	Considerations for HIV Patients	References
Internal fixation (dynamic hip screw/cannulated screws)	Suitable for younger patients with good bone density	A higher malunion risk.	Careful pre-operative assessment of BMD is recommended	[60,80,81]
Hemiarthroplasty	Shorter surgical time; reduced perioperative stress; suitable for older patients with lower functional demands.	Higher risk of periprosthetic joint infection; potential for prosthesis failure in younger patients.	Extended antibiotic prophylaxis and use of antibiotic-impregnated cement are recommended; suitable for patients with moderate life expectancy.	[77,82]
Total Hip Arthroplasty (THA)	Best long-term functional outcomes; suitable for active patients with a longer life expectancy.	Most invasive option; higher risk of post-operative complications, including infection and periprosthetic fractures.	Careful selection between cemented vs. uncemented prosthesis based on BMD; requires diligent post-operative monitoring.	[76,77,83]

### 3.4. Prevention Strategies and Management

Prevention should focus on modifiable lifestyle risk factors, especially nutritional status, toxic habits, promotion of physical activity, and prevention of falls. A proactive approach in prevention is necessary to minimise the consequences of bone loss and morbidity associated with fractures among PLWHIV and, thus, identify potential high-risk factors [84,85].

Smoking is one of the most important lifestyle factors that reduce bone mass [86]. It is almost universally recognised as a risk factor for low BMD and increased fracture risk [87,88], with a Swedish prospective study finding that smokers are at elevated risk for hip fractures [89]. One extensive meta-analysis has found smoking to be a significant modifiable risk factor that should be prioritised by HIV health providers to reduce hip fracture risk when managing PLWHIV [22]. For this reason, there is a need for education on smoking cessation for PLWHIV to reduce their osteoporosis and fracture risk.

The effects of alcohol consumption on the risk of osteoporotic fractures are mediated through both direct, endocrine, metabolic, and nutritional effects that converge on the bone [80]. Several studies have consistently found an increased risk of fractures in men and women with moderate or high alcohol consumption [90,91,92]. Substance use is not uncommon among PLWHIV, as alcohol, cocaine, and opioid use have all been associated with low BMD [93,94]. A 2017 study found that recent drinking was associated with low BMD diagnosis, as was drinking intensity between the first positive HIV test and cART initiation [95].

Management strategies for patients at high risk of fragility fractures include dietary and lifestyle changes [96]. A sedentary lifestyle and behaviours have been linked to bone loss [28], and PLWHIV may be at increased risk due to common associated symptoms (e.g., sleep disturbance, depressive symptoms, fatigue). There is evidence of independent protective effects of both past physical activity and moderate levels of recent physical activity on the risk of hip fracture [97], with one randomised controlled trial (RCT) concluding that PLWHIV could benefit from consistent and long-term engagement in physical activity to prevent bone loss and decrease the risk of fracture [98].

Nutrition is another important factor that affects bone health and hip fracture risk. Poor nutritional status, reflected in inadequate dietary energy intake, reduced serum albumin, and decreased body mass and soft tissues, was significantly associated with a higher risk of subsequent hip fractures [99]. One RCT concluded that it was essential to implement medical treatment by promoting behavioural changes in food intake and lifestyle in the primary prevention of bone disease in chronically infected people [100]. Adequate calcium and vitamin D intake is important to reduce the risk of fracture [101], as vitamin D deficiency has been shown to be highly prevalent in PLWHIV [102]. Therefore, it is very important to continually monitor and optimise patients’ vitamin D and calcium levels, not only because of their positive effects on bone metabolism, but also because one double-blinded RCT found that daily high-dose vitamin D and calcium supplementation over 48 weeks attenuates BMD decline associated with cART initiation by approximately 50% [103].

For patients with high fracture probability, such as those with osteoporosis, bisphosphonate therapy should be considered. Bisphosphonates are regarded as first-line treatments in PLWHIV with osteoporosis because clinical evidence suggests they are well-tolerated, safe, and have a positive BMD response comparable to that of the general population. Alendronate or zoledronic acid is recommended for HIV patients with osteoporosis [104,105]. Other bisphosphonates have not been evaluated in this patient group. PLWHIV should receive 70 mg of alendronate once weekly (with calcium carbonate 1000 mg/vitamin D 400 IU per day). Intravenous zoledronic acid 5 mg yearly can be given as an alternative to alendronate [106]. Bisphosphonate treatment should be monitored closely, as guidelines suggest, because of concerns about the negative effects of long-term suppression of bone turnover, such as osteonecrosis of the jaw and atypical femoral fractures [83]. If the BMD continues to decline following the administration of bisphosphonates, then teriparatide should be considered [106]. One systematic review found that bisphosphonates, especially alendronate, significantly increased bone mineral density at the total hip after 48 and 96 weeks of treatment [107]. The increased fracture risk in people living with HIV results from both the direct effects of the virus and the impact of antiretroviral therapies, as well as associated comorbidities and lifestyle factors. Importantly, evidence indicates that the risk of fractures in PLWHIV has decreased over time. However, despite these improvements, PLWH continue to experience a higher fracture risk compared to the general population. Therefore, ongoing monitoring and individualised management strategies remain essential to mitigate fracture risk in this population [108].

**Figure 3:**
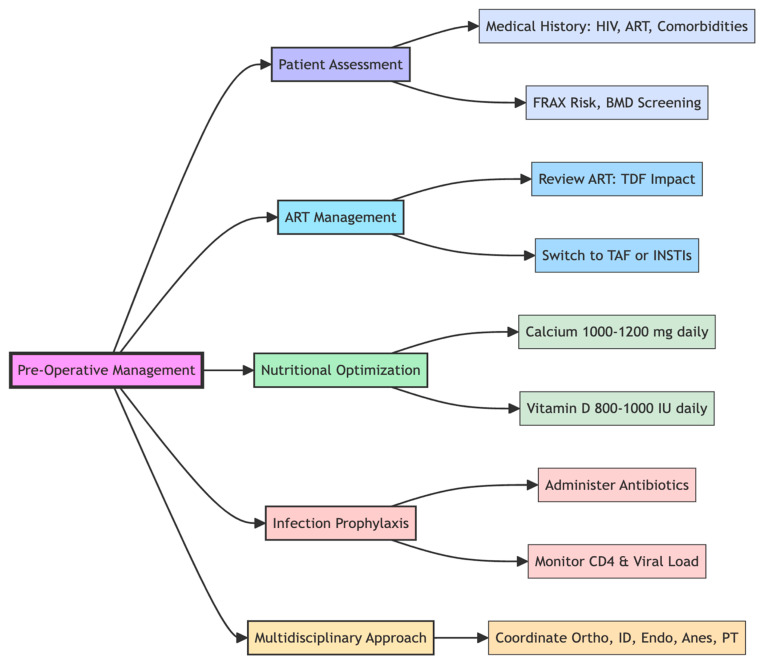
Demonstrating the Pre-operative factors to consider when managing PLWHIV with NOF fractures. Otho: orthopaedic surgeons and orthogeriatric physicians. ID, infectious disease. Endo, endocrinologist. Anes, Anaesthetist.

**Figure 4:**
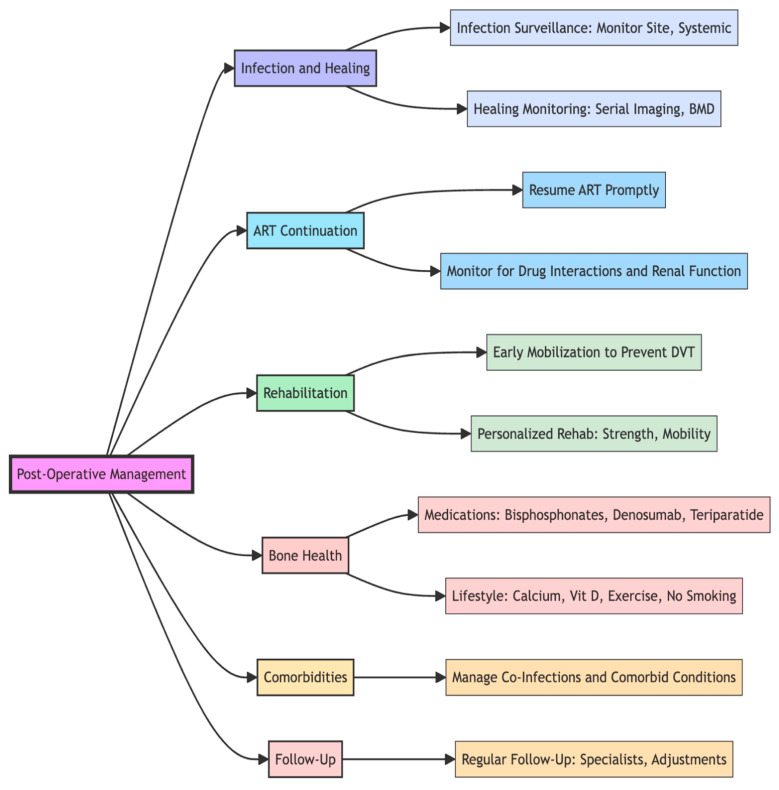
Demonstrating Post-operative considerations in PLWHIV with NOF fractures.

**Figure 5:**
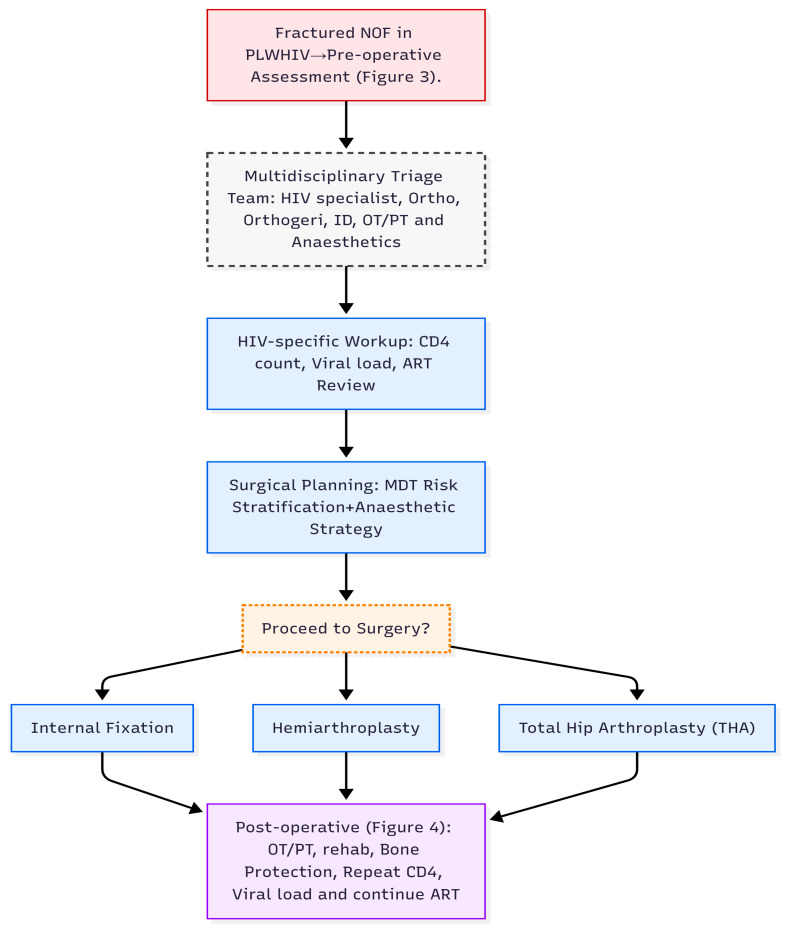
Multidisciplinary care pathway for managing fractured neck of femur (NOF) in people living with HIV (PLWHIV). OT/PT: occupational therapist and physiotherapist, Ortho: Orthopeadic surgeon, Orthogeri: Orthogeriertician, ID: infectious diseases physicians,.

When PLWHIV suffer hip fractures, some earlier publications had suggested that performing hip arthroplasties on these patients put them at risk of higher complications, such as an increased risk of infections and delayed or non-unions of fractures [14,109]. However, Aird et al have found no evidence to suggest that surgical management of fractures in the HIV population should be avoided, stating that surgical fixation and arthroplasties are safe procedures with acceptable outcomes and infection can be risk in those with advanced HIV and low CD4 count [110]. Importantly, HIV infection is associated with an increased risk of post-operative complications; however, this risk is largely dependent on the degree of virological control and immunological function. Patients with uncontrolled viral loads or CD4 counts below 200 cells/mm^3^ are more susceptible to surgical site infections, impaired wound healing, and delayed recovery due to persistent immune dysfunction, chronic inflammation, and nutritional compromise [76,77,78,80]. Conversely, those with well-controlled HIV, defined by suppressed viral loads and CD4 counts above 200–350 cells/mm^3^, generally have post-operative outcomes comparable to HIV-negative individuals [76,77]. Additional perioperative risk factors include anaemia, hypoalbuminaemia, and the complexity of the surgical procedure—for instance, abdominal surgeries are associated with higher complication rates in this population. For most elective orthopaedic procedures, surgery can proceed safely when viral suppression is confirmed and immune function is stable [111]. This highlights the importance of multidisciplinary collaboration, particularly with HIV physicians, to ensure thorough pre-operative optimisation and post-operative monitoring. In summary, while HIV can increase the risk of post-operative complications, effective antiretroviral therapy, immune restoration, and targeted perioperative planning significantly mitigate these risks, enabling safe surgical intervention in this group [111]. A summary of pre- and post-operative considerations for neck of femur fractures in PLWHIV is provided in Table 4, with further detail illustrated in Figure 3, Figure 4 and Figure 5. 

### 3.5. Future Research and the Gaps in Surgical Management of NOF in PLWHIV

The new strategies for bone healing, like the biologic bone grafts, bone morphogenic proteins (BMPs), and bisphosphonates, are promising to improve the delayed fracture healing in PLWHIV; however, there is a need for more trials to establish their effectiveness in this population [122]. The increasing tendency to use minimally invasive surgical techniques may be associated with better results in preventing infection and faster recovery; however, more work is needed to establish the best approaches for immunocompromised patients [93,123]. Perioperative cART management is also an important area that needs further study. More trials are still needed to establish the best time for surgery, the need for cART modification, and the ways of preventing cART-related osteoporosis and fractures [124]. Anaesthesia and perioperative pain management interact with cART, and thus, further study is needed to avoid complications [125]. Post-operative infection control is an important issue as PLWHIV have increased rates of prosthetic joint infections, delayed wound healing, and osteomyelitis. Specific studies on infection prevention protocols and immune status-specific surveillance strategies are important to enhance the surgical practice [126,127].

Future studies should also address long-term functional outcomes, mobility, and re-fracture risk, including high-risk subgroups such as postmenopausal women, those with hepatitis C, fatty liver, or those with chronic kidney disease, in whom bone health is already compromised [128,129]. Novel technologies such as smart implants and artificial intelligence (AI)-based predictive models may hold promise for the future in customised surgical planning and enhanced rehabilitation. More studies are required to determine their effect on recovery, complications, and long-term bone health in PLWHIV [130,131]. Lastly, the cost effectiveness of the various surgical approaches, fixation techniques and the perioperative management in the PLWHIV should be evaluated to guide the healthcare policies and resource management to achieve the best possible patient results at the lowest possible cost [132,133]. Treating these surgical research gaps through a multidisciplinary approach will enhance NOF fracture management in PLWHIV and may lead to better survival, functional recovery, and long-term bone health. Importantly, the metabolic clinic for PLWHIV can help in the prevention and treatment of osteoporosis [134].

## 4. Special Measures and Considerations

Management of fractures of the neck of the femur in people living with HIV (PLWHIV) is a complex clinical challenge that necessitates tailored strategies and special considerations. HIV infection not only affects immune function but also has direct and indirect impacts on bone health, contributing to an increased risk of osteoporosis and fractures, particularly in ageing populations. It is essential to recognise that HIV infection itself is an independent risk factor for reduced bone mineral density (BMD). Several studies have demonstrated a higher prevalence of osteoporosis and a significantly increased risk of fractures in PLWHIV compared to HIV-negative individuals [3,5,9,11,12]. For instance, some studies estimate that approximately 0.6% of PLWHIV may develop osteoporosis attributable solely to HIV infection, without the influence of other confounding factors [6,18,95,105]. While this percentage may seem modest, it underscores a direct biological link between HIV infection and bone loss. This association is largely attributed to the chronic inflammatory state induced by HIV. Persistent immune activation leads to increased production of pro-inflammatory cytokines such as tumour necrosis factor-alpha (TNF-α) and interleukin-6 (IL-6). These cytokines stimulate osteoclast activity, enhancing bone resorption and simultaneously inhibiting osteoblast-mediated bone formation. Moreover, HIV viral proteins can have direct cytopathic effects on bone-forming cells. For example, these proteins may impair osteoblast function and promote osteoclast genesis, further tipping the balance towards bone degradation [6,18,95,105]. HIV also disrupts immune homeostasis by depleting CD4+ T cells, which play a critical role in regulating the RANK/RANKL/OPG pathway—a fundamental signalling axis in bone remodelling. Dysregulation of this pathway accelerates bone turnover, leading to progressive bone loss.

The role of cART is dual in the context of bone health. On one hand, certain ART regimens, particularly those containing tenofovir disoproxil fumarate (TDF), are associated with adverse skeletal effects. TDF is known to cause renal tubular dysfunction, which leads to phosphate wasting (hypophosphatemia) and hypercalciuria. This, in turn, contributes to decreased BMD and increases the risk of osteoporosis [31,32,33]. TDF may also alter vitamin D metabolism, further impairing bone mineralisation. On the other hand, effective and sustained ART can significantly mitigate bone loss by suppressing HIV replication. Suppression of viral load reduces systemic inflammation and immune activation are the two major contributors to HIV-associated osteoporosis. Achieving and maintaining an undetectable viral load is the central goal of HIV treatment, and it is associated with immune stabilisation and improved overall health outcomes. Studies have shown that PLWHIV who maintain undetectable viral loads through consistent cART use demonstrate BMD and fracture risk profiles similar to those of HIV-negative individuals [85,96]. This highlights the protective effect of cART against musculoskeletal complications, particularly when started early and maintained consistently.

When cART is not initiated or maintained adequately, HIV continues to replicate, perpetuating chronic systemic inflammation and ongoing immune dysfunction. These effects accelerate bone turnover, especially through increased bone resorption, leading to reduced BMD over time. Several longitudinal studies have reported that untreated HIV infection is associated with significantly greater bone loss compared to individuals who are virologically suppressed on cART [7,15,17,22,24]. Given the cumulative impact of untreated HIV on bone health, timely initiation and lifelong adherence to cART are crucial in preventing long-term skeletal complications.

Beyond bone fragility and fracture risk, HIV infection also influences surgical outcomes, particularly in patients undergoing orthopaedic procedures such as hip fracture repair. HIV is associated with an increased risk of post-operative complications, including surgical site infections (SSIs), delayed wound healing, systemic infections, and longer hospital stays—especially in individuals with advanced disease, high viral loads, and low CD4 counts [111,112,113]. The immune status of the patient is a major determinant of surgical risk. In individuals with CD4 counts below 200 cells/µL, the risk of post-operative infections and complications is significantly elevated. Additionally, the presence of comorbidities, poor nutritional status, and delayed presentation further contribute to adverse outcomes.

However, individuals with well-controlled HIV infection—those with undetectable viral loads and robust CD4 counts—tend to experience surgical outcomes similar to those of HIV-negative individuals, provided other health parameters are equal. This is because effective cART not only reduces viral burden but also minimises systemic inflammation and the incidence of opportunistic infections, thereby improving wound healing and immune resilience [80,81]. In low-resource settings, HIV physicians face unique challenges in managing musculoskeletal complications in PLWHIV. Limited access to DEXA scans, intermittent cART supply, and structural barriers like transportation, stigma, and food insecurity impede optimal care delivery. In such environments, physicians must prioritise promoting consistent cART adherence, conducting clinical risk assessments for bone loss, providing nutritional support (e.g., calcium and vitamin D), offering patient education on lifestyle factors (e.g., smoking cessation, physical activity), monitoring CD4 counts and viral loads regularly, even in the absence of advanced imaging [42,48]. These pragmatic approaches help reduce the long-term burden of osteoporosis, prevent fragility fractures, and improve surgical outcomes in HIV-positive individuals, even when advanced technology is not available. HIV infection independently increases the risk of osteoporosis, fractures, and post-operative complications, particularly in the absence of effective cART. Poor adherence to cART leads to elevated viral loads and persistent inflammation, accelerating bone loss and increasing vulnerability to surgical complications. Conversely, achieving and maintaining viral suppression through consistent cART use mitigates these risks, bringing bone health and surgical outcomes in line with those of the general population. For HIV care providers, especially in low-resource settings, it is essential to integrate bone health assessments and fracture prevention strategies into routine care. Emphasising adherence to cART, addressing barriers to treatment, and focusing on basic preventive care measures can collectively reduce the incidence of musculoskeletal complications and enhance long-term outcomes for PLWHIV.

## 5. Innovative Concepts

- The potential role of AI in fracture prediction.

AI-guided radiological assessment has demonstrated strong diagnostic performance, making it a reliable and efficient tool for evaluating femoral neck fractures. Its incorporation into routine radiological practice is well justified, especially as a support tool alongside human interpretation, offering faster and more consistent diagnoses. A systematic review and meta-analysis evaluating AI’s diagnostic performance in assessing femoral neck fractures included 11 primary studies with a total of 21,163 radiographs. Using a multilevel random-effects model, this study reported high diagnostic accuracy (0.91), excellent specificity (0.91), and good sensitivity (0.87) [135]. Another systematic review reported AI sensitivity and specificity of 85 % and 87 %, respectively, in detecting neck of femur (NOF) fractures [136]. Notably, a deep learning model utilising a convolutional neural network demonstrated outstanding performance in detecting nondisplaced femoral neck fractures, achieving sensitivity of 97.5 %, specificity of 95.1 %, and overall accuracy of 96.5 % [137]. These findings support the suggestion that AI systems consistently achieve sensitivity and specificity above 85 % for NOF fracture detection;

- New therapeutics like biologic bone grafts and BMPs.

Recent developments in orthobiologics have introduced innovative therapeutic options aimed at improving healing outcomes in femoral neck fractures, particularly in cases with a high risk of complications such as non-union or avascular necrosis. Biologic bone grafts like bone marrow aspirate concentrate (BMAC) are gaining traction for their osteogenic potential. One case report highlighted the effective treatment of a femoral neck non-union in a young patient using BMAC, resulting in successful healing, absence of donor site morbidity, and favourable functional recovery [138]. Bone morphogenetic proteins (BMPs), notably recombinant human BMP-2 (rhBMP-2), have also demonstrated efficacy in promoting bone regeneration. In a retrospective controlled study, patients with displaced femoral neck fractures who received rhBMP-2 in combination with cannulated screw fixation experienced significantly lower rates of non-union and osteonecrosis compared to those treated with screw fixation alone [139]. Furthermore, systematic reviews and meta-analyses of BMP application in long bone non-unions have shown enhanced union rates without an increased risk of infection, supporting their potential utility in more complex fracture cases [140]. Together, these biologic therapies represent a shift towards regenerative and less invasive approaches in the treatment of femoral neck fractures, particularly in younger or high-risk patient populations;

- Smart prosthetic technologies for younger HIV-positive patients.

Younger HIV-positive patients face unique challenges that make smart prosthetic technologies especially valuable for their care. Due to untreated HIV or associated comorbidities, they have an increased risk of infections, peripheral neuropathy, and vascular complications that can lead to limb problems or amputations. Advances in antiretroviral therapy (ART) have enabled many HIV-positive individuals to live longer, more active lives. Younger patients, in particular, benefit significantly from technologies that enhance independence and physical function. Additionally, social stigma can adversely affect their mental health, and smart prosthetics can help improve self-esteem and promote social inclusion. Modern smart prosthetics incorporate advanced sensors that provide real-time feedback on pressure, alignment, and gait, which are critical for preventing injuries in patients with sensory neuropathy. Neural integration technologies enable more natural control and movement, while lightweight, durable materials enhance comfort and wearability. These devices can also be customised to adapt to changes such as weight fluctuations or muscle wasting, which are common in HIV. Integration with smartphones allows healthcare providers to remotely monitor prosthetic performance and detect complications early. Recent developments in smart prosthetic technologies offer promising options for younger HIV-positive patients recovering from femoral neck fractures. Although little work has been performed on smart prosthetics specifically for neck of femur (NOF) fracture implants, related technologies like manometers and Radio Frequency Identification (RFID) for monitoring impact, pressure, and step count are being explored in knee replacements. Similar innovations are planned for hip replacements and may be applicable for NOF patients with HIV, but these are not yet widely available [141,142,143,144].

These advancements aim to improve adaptability, user engagement, and sensory feedback to enhance mobility and independence. Augmented reality (AR)-based prosthetic training platforms, such as HoloPHAM, have demonstrated significant improvements in motor skills—including reach, grasp, and tactile precision—after only 10 days of training, highlighting AR’s potential to accelerate rehabilitation in younger individuals [141]. Furthermore, 5G-enabled, edge-connected prosthetic hands equipped with onboard cameras and real-time data processing enable rapid object detection and responsive grip adjustments with feedback delays under 125 milliseconds, which is particularly beneficial for active younger users [142]. Vision-enabled pediatric prosthetics use wrist-mounted cameras and embedded deep learning models to accurately recognise and grasp objects, achieving near-perfect accuracy while maintaining low cost and power consumption—features that make them ideal for HIV-positive youth in resource-limited settings [143]. Vibro-inertial feedback systems (VIBES), which use vibration actuators embedded in the prosthetic socket, convey tactile information, allowing users to distinguish surface textures, thereby improving safety and intuitive prosthesis use [144]. Collectively, these smart prosthetic innovations support faster rehabilitation, adaptive interaction with the environment, and enhanced sensory integration, making them well-suited for younger HIV-positive patients recovering from femoral neck fractures who require durable, affordable, and responsive prosthetic solutions. However, larger population studies are needed to assess the full potential benefits of these technologies for younger people living with HIV. Overall, smart prosthetics can enhance mobility, physical health, mental well-being, and social participation. Still, challenges such as high costs, limited access in resource-poor areas, the need for integrated HIV and prosthetic care, and psychosocial support remain important considerations.

### Strengths and Limitations

There are several weaknesses that can be associated with this review: First, there are very few large-scale, good-quality randomised controlled trials that have been conducted to manage NOF fractures in PLWHIV. Furthermore, the long-term results of surgical treatments in PLWHIV are unknown, and there is limited data in terms of the survival of the implant, the functional outcome, and the rates of revision surgery. Future work should include prospective studies of the effectiveness of surgical methods (total hip arthroplasty, hemiarthroplasty, internal fixation) in HIV positive patients. The use of different cART regimens, the immune status, and the presence of co-morbidities of PLWHIV make it difficult to identify specific surgical recommendations. The heterogeneity in fracture fixation modalities (internal fixation and arthroplasty), perioperative antimicrobial prophylaxis, and protocols for early post-operative care and rehabilitation measures also makes it difficult to compare the results of one study to another. This narrative review compiles a large amount of information on the surgical treatment of neck of femur fractures in people living with HIV (PLWHIV), the challenges encountered in the perioperative period, the surgical techniques used, the complications seen in the post-operative period, and the long-term results. It discusses the special concerns in HIV-positive patients, which include bone strength, cART-related metabolic abnormalities, and increased risk of infection, which are crucial in the management of the patient. The major advantage of this review is that it presents a comprehensive overview of the existing research on operative procedures, fixation techniques, and post-operative care in HIV positive patients. This review is valuable for orthopaedic surgeons, trauma doctors, HIV specialists, and the teams responsible for the care of patients before, during, and after surgery from the points of view of orthopaedic surgery, infectious diseases, and geriatrics. Furthermore, this review explores the importance of care during the perioperative period, prosthesis choice, and rehabilitation aspects to aid in the decision-making process and improve the care of patients with PLWHIV with NOF fractures.

## 6. Conclusions

Neck of femur fractures in people living with HIV (PLWHIV) represent a significant and evolving clinical challenge, particularly as survival improves and the HIV-positive population ages. This narrative review demonstrates that PLWHIV have an elevated risk of such fractures due to a combination of cART-related bone demineralisation, chronic inflammation, and comorbidities. Although surgery remains the mainstay of treatment, post-operative outcomes depend heavily on viral suppression, CD4 count, comorbidity burden, and fracture type. Notably, evidence suggests that virally suppressed individuals with stable immune function can undergo surgical management with complication rates similar to HIV-negative patients. In contrast, those with uncontrolled viraemia or advanced immunosuppression face a higher risk of wound infections, delayed healing, and implant failure. We have incorporated a broader literature base to support these conclusions, and while this is a narrative review, the expanded evidence highlights the need for personalised, multidisciplinary care and improved data collection in this patient group. Future prospective studies are required to define optimal surgical strategies, rehabilitation pathways, and long-term outcomes for PLWHIV presenting with NOF fractures (Figure 5).

## Figures and Tables

**Table 1:** Showing high prevalence of NOF among PLWHIV in different continents.

Region	Key Findings	Comparison with HIV-Negative Individuals	References
North America	PLWHIV are twice as likely to sustain fractures, including NOF. HOPS data confirms higher NOF incidence.	NOFs among HIV-positive individuals can be common association.	[10,11]
Europe	Higher incidence of femoral neck fractures in HIV-positive individuals.	Highlights need for preventive strategies in PLWHIV.	[14,15]
Africa	South African cohort study found a higher prevalence of NOFs in PLWHIV.	Associated with high HIV prevalence and widespread tenofovir use.	[16]
Asia	Increasing prevalence of femur neck fractures in PLWHIV, particularly in ageing populations.	Higher NOF prevalence and low bone mineral density in PLWHIV.	[17]

**Table 4:** Demonstrating Pre- and Post-operative considerations for NOF fractures in PLWHIV.

Category	Pre-Operative	Post-Operative	References
HIV and Immune Status	Check viral load (<6M), CD4 (<12M). Delay surgery if CD4 < 200. Assess CVD, CKD (tenofovir), HBV/HCV.	Recheck CD4/viral load if cART is delayed. High infection risk (CD4 < 200).	[112,113]
Nutrition and Bone	Screen for osteoporosis, anaemia, vitamin D. Optimise intake.	High-protein diet, continue calcium/vitamin D.	[114,115,116]
cART and Drug Interactions	Continue cART unless contraindicated. Avoid PI-anaesthetic interactions. Restart ASAP if interrupted.	Resume early, avoid CYP3A4 inhibitors (rifampin, ketoconazole).	[112,117]
Infection Risk	Consider antibiotic prophylaxis (high-risk surgeries). Higher pneumonia, wound, UTI risk.	Monitor for MRSA and fungal infections, adjust antibiotics (CD4-based).	[118,119]
Anaesthesia	Avoid CYP3A4-metabolised drugs (midazolam, fentanyl). Prefer regional.	Monitor emergence, adjust pain meds to prevent cART interactions.	[117,120]
Multidisciplinary	Pre-op: ID, anaesthesia, surgery, pharmacist (cART review).	Post-op: ID follow-up (1–2W), physio, dietitian support.	[74]
Psychosocial	Screen for depression and adherence barriers.	Ensure cART adherence, mental health support.	[121]

## Data Availability

No new data were created or analyzed in this study. Data sharing is not applicable to this article.
